# Sphere-forming cells from peripheral cornea demonstrate the ability to repopulate the ocular surface

**DOI:** 10.1186/s13287-016-0339-7

**Published:** 2016-06-01

**Authors:** Jeremy John Mathan, Salim Ismail, Jennifer Jane McGhee, Charles Ninian John McGhee, Trevor Sherwin

**Affiliations:** Department of Ophthalmology, New Zealand National Eye Centre, Faculty of Medical and Health Sciences, The University of Auckland, Private Bag 92019, Auckland, 1010 New Zealand

**Keywords:** Cell culture, Cornea, Holoclone, Immunocytochemistry, Quantitative PCR, Spheroid

## Abstract

**Background:**

The limbus forms the outer rim of the cornea at the corneoscleral junction and harbours a population of stem cells for corneal maintenance. Injuries to the limbus, through disease or accidents such as chemical injuries or burns, may lead to significant visual impairment due to depletion of the native stem cells of the tissue.

**Methods:**

Sphere-forming cells were isolated from peripheral cornea for potential use as transplantable elements for limbal stem cell repopulation and limbal reconstruction. Immunocytochemistry, live cell imaging and quantitative PCR were used to characterize spheres and elucidate activity post implantation into human cadaveric corneal tissue.

**Results:**

Spheres stained positively for stem cell markers ∆NP63α, ABCG2 and ABCB5 as well as the basal limbal marker and putative niche marker, notch 1. In addition, spheres also stained positively for markers of corneal cells, vimentin, keratin 3, keratocan and laminin, indicating a heterogeneous mix of stromal and epithelial-origin cells. Upon implantation into decellularized corneoscleral tissue, 3D, polarized and radially orientated cell migration with cell proliferation was observed. Cells migrated out from the spheres and repopulated the entire corneal surface over 14 days. Post-implantation analysis revealed qualitative evidence of stem, stromal and epithelial cell markers while quantitative PCR showed a quantitative reduction in keratocan and laminin expression indicative of an enhanced progenitor cell response. Proliferation, quantified by PCNA expression, significantly increased at 4 days subsequently followed by a decrease at day 7 post implantation.

**Conclusion:**

These observations suggest great promise for the potential of peripheral corneal spheres as transplantable units for corneal repair, targeting ocular surface regeneration and stem cell repopulation.

## Background

The anterior ocular surface is a continuous sheet of tissue that consists of the transparent cornea and the more peripherally located conjunctiva overlying the opaque, white, sclera. The cornea transitions into the sclera at the zone known as the corneoscleral junction or the limbus. The corneal limbus, the in-vivo location of corneal epithelial stem cells, is the transitional region between the cornea and conjunctiva/sclera. Anatomically, the basal layer of the limbal epithelium appears corrugated because it is arranged in rete pegs (finger-like projections of the epithelium into the stroma below), also named interpalisadal rete ridges, with the upward projections of the stroma termed the palisades of Vogt [1–4]. The longstanding view has been that these rete ridges harbour the cells for corneal maintenance. Indeed, the limbal stem cells divide to give rise to progeny which maintain the structure and function of the cornea [5].

The limbal epithelial crypts (long extensions of the interpalisadal rete ridges) described relatively recently have also been shown to be potential reservoirs of stem cells [6]. Various injurious processes such as intrinsic diseases, chemical injuries and thermal burns [7] can damage the limbal environment and deplete the stem cell population therein, thus impairing the regenerative capacity of the cornea leading to redness and pain, persisting epithelial defects, corneal vascularization, conjunctivalization and ultimately severe visual impairment or blindness. Severe depletion of stem cells within the limbal environment can lead to a condition known as limbal stem cell deficiency (LSCD).

The stem cell niche or microenvironment is responsible for maintaining the stem cell phenotype and directing differentiating cells along a corneal cell differentiation pathway. The importance of the stem cell niche—which includes the cells of the tissue that surround the stem cells along with the signalling molecules they secrete [8–10] as well as the extracellular matrix in directing stem cell differentiation within tissue [11, 12]—is well established, especially within the study of haematopoietic stem cells.

The limbal microenvironment has been shown to be capable of directing the programming of hair follicle stem cells toward a more cornea-like phenotype [13]. In mice, pluripotent stem cells were stimulated to acquire corneal epithelial morphology when exposed to the limbal stromal environment [14]. It is clear that limbal stromal cells are important in maintaining both the limbal and corneal epithelial phenotype. Additionally, the observation that differentiated cells of the central cornea can be stimulated to de-differentiate and develop a dermal phenotype when placed in that environment [15] underscores the need to consider stem cells in context of their immediate microenvironment.

The limbal stroma is an integral part of this microenvironment. Firstly, it is noted that limbal stromal fibroblasts cross the basement membrane and adhere to stem and progenitor cells in the basal epithelium above [16]. In-vitro exposure of limbal epithelial cells to the cells of the limbal stroma in co-culturing experiments also appears to increase certain indicators of stemness. This is manifested as an increase in the expansion capacity of cultured limbal epithelial cells [17] and an increase in putative stem cell markers such as p63 and ABCG2 [18], when compared with cells not cultured in the presence of limbal stromal cells.

Secondly, the limbal stromal environment appears to have the ability to discourage the presence of cells foreign to the niche and promote the presence of cells usually found within the niche. This is seen through the restoration of transparency in previously diseased, conjunctivalized and opacified, corneal epithelia when exposed to limbal stroma conditioned medium [19]. Observations of the loss of the limbal crypt structure being correlated with a decline in stemness of cells [20], the greater success in culturing stem cells in 3D compared with 2D culture systems [21] and the improved clonality when cellular connections are maintained [22, 23] have lent support for the importance of the 3D extracellular matrix structure in maintaining the stemness of limbal epithelial cells in culture.

The sphere-forming assay is one approach for enriching cell populations with stem cells in vitro. This is a cell culture method that involves incubating cells in a serum-free medium supplemented with growth factors which selectively encourage the survival of stem and progenitor cells [24, 25]. Cells cultured from mice gradually form well-defined spherical entities, ranging from 50 to 150 μm and connected to each other via gap and adherence junctions, over a period of 7 days [24]. The sphere-forming assay has also been successfully used to generate spheres from cells isolated from human ocular tissue [26–28]. These spheres, although enriched with stem cells, are composed of a heterogeneous mix of cells within the continuum from stem to differentiated cells [29]. For peripheral corneal spheres, this is advantageous because this method of stem cell enrichment not only mimics the in-vivo heterogeneity of the natural limbus but also achieves this within a 3D format which allows for intercellular attachment.

Stem cell-enriched sphere-based therapy remains a promising treatment approach for corneal stem cell repopulation. We have previously shown human peripheral corneal spheres to be dynamic entities that demonstrate polarity and directed cell migration and are capable of initiating a wound healing response to injury in vitro [26]. The in-situ behaviour of these peripheral corneal spheres, however, is yet to be characterized. Here we advance on our previous work as we further characterize human peripheral corneal spheres in vitro, implant them into human donor corneoscleral rims and present evidence to show their capacity for in-situ ocular surface repopulation.

## Methods

### Human tissue

Fresh and frozen human corneoscleral rims and frozen human amniotic membrane were obtained from the New Zealand National Eye Bank with approval from the Northern X Regional Ethics Committee. Consent for human corneal tissue use for the purposes of research is attained prior to eye banking.

### Sphere formation and culture

Spheres were generated from human limbal tissue using a cell extraction process and the sphere-forming assay essentially as described previously [30]. A single, entire human donor corneoscleral rim provided by the New Zealand National Eye bank was used to form a sphere batch. The clear cornea component of the rim was used, excising as close to the limbus as possible but excluding the sclera. Briefly, this process initially involved the removal of the corneal endothelium. The corneoscleral region was excised and the tissue was de-epithelialized using a scalpel blade. The tissue was then incubated in 1.2 U/ml dispase II for 40 min at 37 °C. Subsequently, the tissue was incubated in 2 mg/ml collagenase and 0.5 mg/ml hyaluronidase overnight at 37 °C on a shaker. This extract was strained using a 40-μm strainer to remove undigested material (BD Biosciences, Franklin Lakes, NJ, USA). The filtrate was centrifuged for 7 min at 405 × *g* and the cell pellet washed with phosphate-buffered saline (PBS). The yield of cells from such an isolation is between 5 × 10^4^ and 1 × 10^5^. Cells were suspended in supplemented Neurobasal-A medium (Neurobasal-A (Life Technologies, Grand Island, NY, USA) with 2 ng/ml epidermal growth factor (Abacus ALS, Auckland, New Zealand), 1 ng/ml fibroblastic growth factor 2 (Abacus ALS), 1 × B27 (50 × stock; Life Technologies), 1 × N2 (100 × stock; Life Technologies), 2 μg/ml heparin (Sigma Aldrich, St Louis, MO, USA), 2 mM GlutaMAX™ Supplement (Life Technologies), 1 × Antibiotic–Antimycotic (Anti-Anti; Life Technologies)) and seeded into wells containing sterile glass coverslips on the well surface. Cells were maintained in culture in humidified incubators at 37 °C in an atmosphere containing 5 % CO_2_ to facilitate sphere formation. Fifty per cent of the spent medium was removed and replaced twice weekly. Over the course of 1–2 weeks, cells become adherent to the glass coverslip and aggregate into sphere-like structures. Spheres are maintained in this culture protocol for use in experiments after at least 1 month in sphere culture conditions. This process selects for and concentrates less differentiated cells existing within tissue into sphere-like structures.

### Preparation of in-vitro and in-situ sphere attachment surfaces

Poly-l-lysine (Sigma-Aldrich)-coated coverslips were prepared for the immobilization of spheres for immunostaining according to the manufacturer’s recommendations. A collagen-coated surface to stimulate sphere cell migration was prepared using Collagen I Rat Protein, Tail (Life Technologies).

Human corneoscleral rims, obtained post surgery and freeze-stored at –80°C for longer than 3 months, were subject to a total of three freeze–thaw cycles to ensure the effective depopulation of the native cells prior to implantation. In a Gelman HLF-120 horizontal laminar flow cabinet and using a Zeiss SV6 Binocular Stereo microscope, frozen and stored human corneoscleral rims were thawed and cut into one-eighth segments using straight scissors. Microsurgical techniques for the implantation of spheres into the epithelial side of the tissue were explored and developed using an ophthalmic surgical microscope (Carl Zeiss, Oberkochen, Germany), a 3.75-mm Short Cut blade (Alcon, Mt Wellington, New Zealand), a Feather MicroScalpel (pfmmedical, Cologne, Germany) and fine forceps.

Spheres implanted onto collagen-coated coverslips and in tissue were incubated with standard culture medium: MEM (1×) GlutaMAX (Life Technologies) supplemented with 10% fetal calf serum and Anti-Anti (Life Technologies). Cell proliferation was identified using Click-iT® EdU Alexa Fluor® 594 Imaging Kit (Life Technologies) by supplementing standard culture medium with 5-ethynyl-2′-deoxyuridine (EDU) at a concentration of 10 μM.

To assess the viability of spheres and implanted cells in tissue, LIVE/DEAD® 2 μM calcein AM and 4 μM ethidium homodimer-1 (Life Technologies) in standard culture medium was used.

### Immunocytochemistry

Immobilized spheres and whole-tissue implants were fixed using 4% paraformaldehyde (PFA) (Sigma Aldrich) in PBS and permeabilized in methanol for 10 min at –20 °C. To block non-specific antibody binding, samples were incubated for 2 h on a shaker in 100 mM glycine, 0.1 % Triton X-100 (Serva Electrophoresis GmbH, Heidelberg, Germany), 10 % normal goat serum (NGS; Life Technologies) in PBS. Where relevant, samples were then incubated in the Click-iT® EDU reaction cocktail as per the manufacturer’s recommendations for 30 min on a shaker. Samples were then washed in PBS with 3% bovine serum albumin (PBS-B) and incubated overnight at 4 °C with primary antibody prepared in PBS-B + 0.5 % Triton X-100. The primary antibodies used were as follows: anti-ABCB5 at 1:125 (#HPA026975; Sigma Aldrich), anti-∆Np63 at 1:200 (private order; PickCell Laboratories, Amsterdam, Netherlands), anti-ABCG2 at 1:25 (#14-8888; eBioscience, San Diego, CA, USA), anti-Notch1 at 1:500 (#MS-1339; Thermo Scientific, Waltham, MA, USA ), anti-Keratocan at 1:100 (#Sc66941; Santa Cruz, Dallas, TX, USA), anti-Vimentin at 1:200 (#V6630; Novocastra, Newcastle, United Kingdom) and anti-Keratin K3/K76 at 1:50 (#CBL218; Millipore, Billerica, MA, USA). Samples were treated with a 1-h secondary antibody incubation prior to rinsing with PBS and counterstaining with 0.1 μg/ml 4′,6-diamidino-2-phenylindole (DAPI). Secondary antibodies were used at a 1:350 dilution and are as follows: goat anti-mouse Alexa488 (#A11029; Molecular Probes, Eugene, OR, USA) and goat anti-rabbit Alexa488 (#A11034; Molecular Probes).

Tissue sections were fixed with 2.5 % PFA, and then incubated with 2 mg/ml testicular hyaluronidase (Sigma Aldrich) in Tris–HCl for 1 h at 37 °C in a humidity chamber. Samples were permeabilized in methanol at –20 °C for 20 min. Sections were then treated with 20 mM glycine in Tris saline buffer (TSB) for 30 min and blocked in 2 % NGS with 0.1 % Triton X-100 in TSB for 30 min at room temperature. Primary and secondary antibodies were prepared in TSB and incubated and counterstained as already described except that secondary antibodies were incubated for 2 h at room temperature.

For all experiments, controls used included secondary antibody only, primary antibody only and no antibody. For spheres implanted in tissue, non-implanted tissue only stained with both primary and secondary antibodies was used as a control.

### Microscopy and imaging

Bright-field images, assessed using the SV6 Binocular Stereo microscope (Carl Zeiss), were captured using a NIKON Digital sight DS-UI camera (NIKON CORPORATION, Tokyo, Japan). Phase-contrast and fluorescence microscopy was performed using the following microscopes: Leica DM IL inverted contrasting microscope (Leica Microsystems, Wetzlar, Germany), 4× magnification 0.1 aperture, C PLAN with Leica Application Suite Version 4.4.0 Build 454; and Leica DM-RA upright fluorescence microscope (Leica Microsystems), 5× magnification 0.15 aperture, HC PL Fluotar and 40× magnification, 1.00 aperture, PL FLUOTAR Oil PH3 with NIS-Elements Br Microscope Imaging Software version 3.0 and images captured using the NIKON Digital sight DS-UI camera (NIKON). Confocal fluorescence microscopy was performed using the Olympus FV 1000 Confocal laser scanning microscope (Olympus America, Center Valley, PA, USA), 20× magnification, 0.75 aperture U Plan S APO and 60× magnification, 1.35 aperture U Plan S APO oil with the FV10-ASW version 0.4.00 image capture and analysis software.

### Quantitative PCR

RNA isolation from pre-implanted and post-implanted sphere cells was performed using the TRIzol® method (Life Technologies) according to the manufacturer’s protocol. DNA digestion was performed using DNase I (RNase-free) (#MO303S; New England Biolabs, Ipswich, MA, USA) according to the manufacturer’s recommendations although the incubation time was increased to 2 h to ensure complete genomic DNA removal. cDNA synthesis was performed in a Peltier Thermal Cycler PTC-200 (MJ Research, Waltham, MA, USA) using 1× SuperScript® VILO™ cDNA Synthesis Kit (#11754050; Life Technologies) according to the manufacturer’s recommendations. Successful cDNA synthesis quality control was performed by PCR using β-actin and GAPDH primers presented in Table 1 and products were analysed by agarose gel electrophoresis detection with gel red dye (Biotium, Hayward, CA, USA). Gels were imaged using the Gel Doc™ EZ Imager (Bio-Rad Laboratories, Hercules, CA, USA) with Image Lab™ software version 5.0 (Bio-Rad Laboratories).

**Table 1:** Quantitative PCR primers used in this study

Product of interest	Primer sets	Size of cDNA amplicon (base pairs)	Size of gDNA amplicon (base pairs)
ATP-binding cassette, sub-family G (WHITE), member 2 (ABCG2)	(F): CCTGAGATCCTGAGCCTTTG (R): AAGCCATTGGTGTTTCCTTG	124	184,966
Proliferating cell nuclear antigen (PCNA)	(F): GGCGTGAACCTCACCAGTAT (R): TTCTCCTGGTTTGGTGCTTC	125	0
Vimentin (VIM)	(F): CCAAACTTTTCCTCCCTGAACC (R): GTGATGCTGAGAAGTTTCGTTGA	141	1395
Laminin, alpha 1 (LAMA1)	(F): ACACCGGGAAGTGTCTGAAC (R): GCTTGAGGAGCACCTTTCAC	239	0
Keratocan (KERA)	(F): ATCTGCAGCACCTTCACCTT (R): CATTGGAATTGGTGGTTTGA	167	4043
Actin beta (β-actin)	(F): AACTCCATCATGAAGTGTGACG (R): GATCCACATCTGCTGGAAG	234	345
Glycreraldehyde-3-phosphate dehydrogenase (GAPDH)	(F): CTGACTTCAACAGCGACACC (R): CCCTGTTGCTGTAGCCAAAT	120	224
ABCB5	(F): TACTCTTCCCACTGCCATTG (R): CAATTATCCATCAAGACCATCTATCAAG (Probe): 56-FAM/CCGACCAAG/ZEN/GCGACTGTCTCT/3IABkFQ	106	0
p63alpha	(F): GGGTCGTGAAATAGTCCAGAC (R): CATCCACCTCCCACTGC (Probe): 56-FAM/CACCTCCGT/ZEN/ATCCCACAGATTGCA/3IABkFQ	108	0
alphaSMA	(F): CTGTTGTAGGTGGTTTCATGGA (R): AGAGTTACGAGTTGCCTGATG (Probe): 56-FAM/AGACCCTGT/ZEN/TCCAGCCATCCTTC/3IABkFQ	131	0
Notch1	(F): ACAGATGCCCAGTGAAGC (R): CGAGGTCAACACAGACGAG	112	1289

All primers and probes assays were purchased from Integrated DNA Technologies (Singapore)

*F* forward, *R* reverse

Quantitative PCR was performed using the Lightcycler® 480 SYBR Green I Master mix or the Lightcycler® 480 Probes Master mix (Roche, Auckland New Zealand) as appropriate according to the manufacturer’s recommendations using the primer sets (or probe-based assays) presented in Table 1 and purchased from Integrated DNA Technologies (IDT, Singapore). Template cDNA synthesized from an equivalent of 1 ng/μl of RNA was used per 10 μl reaction.

All quantitative PCR experiments were conducted in a Rotor-Gene™ 6000 (Corbett Life Science, Sydney, Australia) and analysed using the Rotor-Gene Q pure detection software version 2.1.0 (Build 9). Cycling conditions for SYBR green detection included an initial activation for 10 min at 95 °C and 40 cycles of 95 °C for 10 sec, 60 °C for 15 sec and 72 °C for 20 sec with detection on the green channel at this third step, while cycling conditions for probe-based assays consisted of initial activation for 10 min at 95 °C and 40 cycles of 95 °C for 10 sec and 58 °C for 45 sec with detection on the green channel at this second step. Quantification of gene expression was performed by measuring 10-fold serial dilutions of purified amplicons with known copy numbers. Two replicates of triplicate measurements for each gene of interest were performed for pre-implantation and post-implantation spheres. The geometric mean for β-actin and GAPDH, the two most stably expressed reference genes across samples, as determined by the statistical algorithm NormFinder, was used for normalization. Following recommendations, non-detects in data were treated as missing values in order to reduce bias [31].

### Statistical analysis

Data collection and statistical analysis were performed using Microsoft Excel 2010 version 14.0.7143.5000 (Microsoft Corporation, Washington, DC, USA) and Statistical Package for the Social Sciences (SPSS) v21.0 (IBM, New York, USA). One-way ANOVAs with Tukey post-hoc tests were conducted to analyse significance of inter-group variation in gene expression. *p* < 0.05 was considered significant.

## Results

### In-vitro sphere characterization

**Fig. 1:**
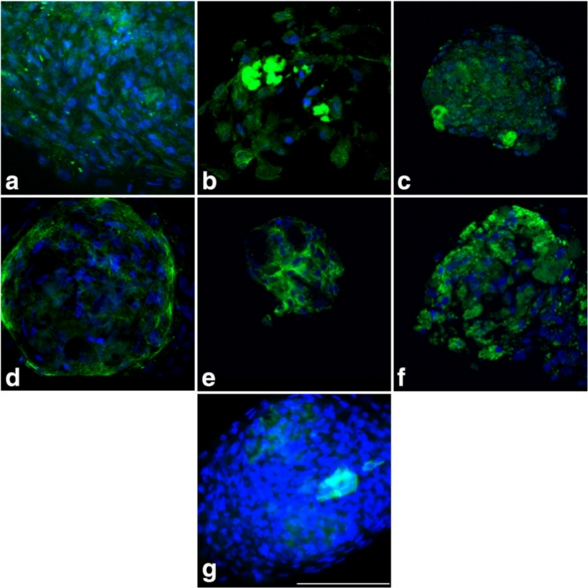
Immunostaining of peripheral corneal spheres reveals expression of putative stem cell and niche markers. Spheres were imaged at 60× objective magnification by confocal microscopy and labelled with antibodies (*green* signal) for ∆Np63α **a**, ABCG2 **b**, ABCB5 **c**, notch 1 **d**, laminin **e** and keratocan **f**. Representative image of the secondary-antibody-only control **g**. *Blue* signals represent DAPI staining of sphere cell nuclei. Scale bar = 100 μm (Colour figure online)

Peripheral corneal spheres were initially characterized in vitro to confirm they were stem-cell enriched and possessed the ability to respond to a collagen matrix with cell migration and division. Spheres immobilized on poly-l-lysine-coated dishes stained positively for putative limbal stem cell markers ∆Np63α, ABCG2 and the recently proposed limbal stem cell marker ABCB5 [32] (Fig. 1a–c) when compared with the background fluorescence emitted by the secondary antibody only (Fig. 1g), primary antibody only and no antibody controls (not shown). Hyper-fluorescent debris were noted at the centre of both test and control spheres. These artefactual signals are commonly observed in the imaging of spheres and were not considered when analysing the true positive signal.

Spheres also stained positively for the limbal basal epithelial marker notch 1 and the corneal extracellular matrix markers laminin and keratocan (Fig. 1d–f). Laminin staining appeared as hyperfluorescent streaks resembling portions of a basement membrane. Notch 1 and, to a lesser extent, keratocan staining was strongly concentrated in the outer region of the sphere compared with the central sphere, contrasting with the localization of the stem cell markers and laminin (Fig. 1d–f).

**Fig. 2:**
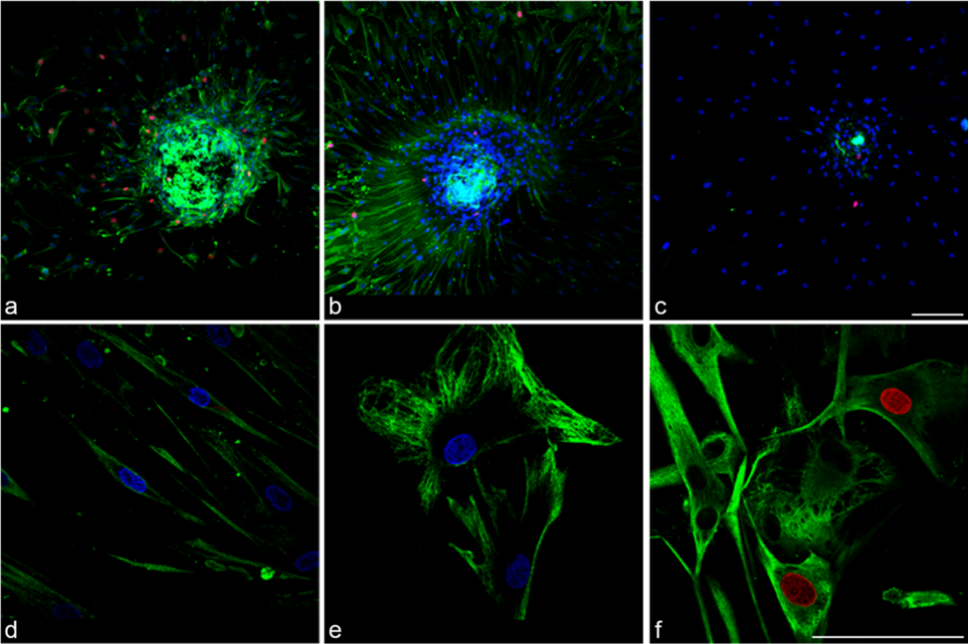
Immunostaining of peripheral corneal spheres stimulated by collagen I substrate reveals expression of cell proliferation and differentiation markers. Spheres were imaged at 20× objective magnification by confocal microscopy and labelled with antibodies (*green* signal) for keratin 3 **a** and vimentin **b**. The secondary-antibody-only control **c** did not show this signal. *Blue* signals represent DAPI staining of DNA within cell nuclei. Red signals represent EDU-incorporated cell nuclei indicative of proliferating cells. At 60× magnification, vimentin-positive migrated cells proximal to the sphere **d** show a different morphology compared with distal cells **e** and some show positivity for the EDU cell proliferation marker (*red*) **f**. Scale bar =100 μm (Colour figure online)

Spheres placed on collagen-coated coverslips and incubated in serum-containing medium showed a radial pattern of cell migration outward from the central sphere after 4 days in culture (Fig. 2a–c). Migrating cells stained positively for the differentiated corneal epithelial cell and corneal stromal markers, keratin 3 (Fig. 2a) and vimentin (Fig. 2b) respectively. While keratin 3 staining was stronger in the central sphere in comparison with cells migrating peripherally, vimentin showed a preferential staining pattern in migratory cells. EDU-incorporated cell nuclei (indicating proliferating cells) are detected both within the central sphere and in cells migrating peripherally and are observed to co-localize with both the differentiation marker keratin 3 as well as the mesenchymal marker vimentin. Vimentin-positive cells immediately migrating out from the sphere had characteristic spindle morphology with long tapering, bidirectional cytoplasmic exstensions (Fig. 2d). Cells are more tightly packed and fibres are radially oriented out from the sphere. In contrast, cells at the leading edge of the migratory wave showed multi-directional cytoplasmic extensions giving the cell a large and spread out appearance (Fig. 2e, f).

### Sphere implantation

**Fig. 3:**
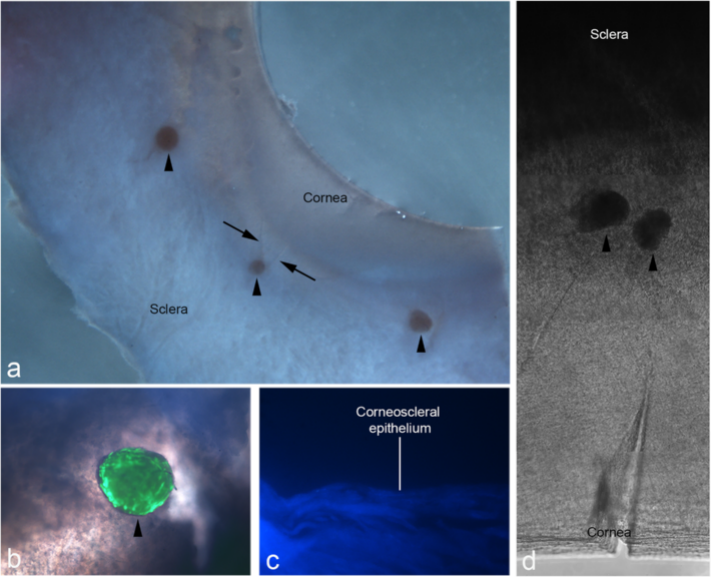
Implantation of peripheral corneal spheres into donor corneoscleral rims. Spheres (*arrowheads*) were implanted into wedge-shaped incisions made at the limbal region. Under stereomicroscopy, the corneoscleral rim with incisions (*arrows*) and implanted spheres can be clearly visualized **a**. Combined phase-contrast and fluorescence microscopy show an implanted sphere stained positively for live cells with LIVE/DEAD® stain **b**. This signal is confined to the sphere and not detected in the surrounding tissue. A 40× DAPI-stained 10-μm thick cross-section of frozen-stored corneoscleral rim confirmed the absence of DAPI-positive cell nuclei prior to implantation **c**. Through phase-contrast microscopy, the position of the spheres in the semi-transparent region of tissue is shown **d**

Frozen-stored corneoscleral tissue was used as a model of limbal stem cell deficiency. It was deemed to possess no viable cells as shown by the absence of DAPI-positive nuclei in sections of the tissue (Fig. 3c). A wedge-shaped ‘trough’ created by incisions subtending an angle of ~60^o^ with the base of the wedge facing the cornea and the apex facing the sclera exposed the limbus and allowed for the placement of spheres (Fig. 3a). The scleral border of the limbus, for incisions, was visually approximated to be in the region where the tissue was neither completely clear nor completely opaque. Phase-contrast microscopy of implanted spheres showed opaque spheres within the semi-transparent tissue (Fig. 3d). LIVE/DEAD® staining showed a strong green fluorescent signal (for live cells) confined within implanted spheres in all experiments (Fig. 3b). Implanted spheres remained in place for the duration of each experiment, which was up to 241 h post implantation, despite being submerged in culture medium and subject to physical agitation during handling.

**Fig. 4:**
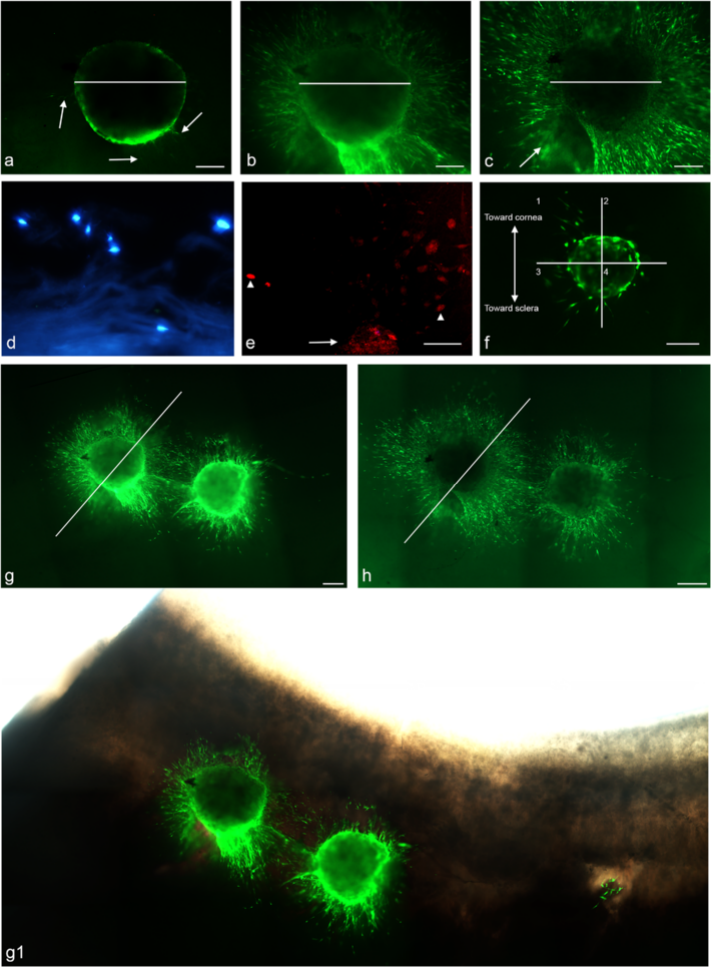
Implantation of peripheral corneal spheres into corneoscleral tissue results in cell migration, inter-sphere interaction and polarized outgrowth. LIVE/DEAD® staining of sphere-implanted tissue showed a *green* (live cell) fluorescent signal in the outline of the sphere with the beginnings of cell migration and minimal tissue staining (*arrows*) at 25 h **a**. An increase in live cell migration is observed in tissue over time from 72 h **b** to 217 h **c**. *Arrow* indicates a blurred region of tissue staining in a different plane of focus **c**. *White* lines at all three time points are of equal lengths showing a decline in sphere diameter from 72 h **b** to 217 h **c**. In cross-section, cell nuclei labelled using DAPI (*blue*) are dispersed **d**. Confocal imaging of EDU staining shows *red* signals in the sphere (*arrow*) and in migrated cell nuclei (*arrowhead*) **e**. Polarized cell migration is evidenced in implanted spheres **f**. Two spheres implanted adjacent to each other in tissue are imaged first at 72 h **g, g1** and subsequently at 217 h **h**. Here, cells migrate from each sphere in the direction of each other. Migration on the right of the diagonal line appears to have increased from 72 h to 217 h more so than on the left of the diagonal line. Montage imaging of the spheres at 72 h post implantation with light microscope image overlay shows the position of spheres at the limbal region of corneoscleral rims **g1**. Scale bar = 100 μm (Colour figure online)

When compared with freshly implanted spheres at 0 h (Fig. 3b), evidence of live cell migration was detected at 25 h post implantation (Fig. 4a). A pronounced increase in cell migration was detected at 72 h post implantation (Fig. 4b) when cells had migrated radially out from the sphere and in multiple focal planes. More extensive cell migration was observed at 217 h post implantation (Fig. 4c). The longest horizontal (visually approximated) diameter of the sphere decreased from 72 to 217 h. In cross-section, sphere cell nuclei appeared dispersed rather than congregating as a single spherical entity (Fig. 4d). Positive EDU labelling was apparent within the sphere and in cells migrating out from the sphere (Fig. 4e), indicating a proliferative response.

Implanted spheres showed active organization of cell migration patterns with 7/11 (63.6 %) spheres exhibiting directed cell migration. This is demonstrated in Fig. 4f by the relative absence of migrating cells in quadrants 2 and 4 and the presence of cells in quadrants 1 and 3 observed in the early stages of cell migration and persisting up to 144 h (not shown).

Spheres placed adjacent to each other showed a cell migration pattern from each sphere towards as well as away from each other. Qualitatively, there appeared to be a disproportionate increase in cell migration from one sphere (left sphere, Fig. 4g and g1) in the direction of the neighbouring sphere (right sphere, Fig. 4g and g1) over time (Fig. 4h).

### Ocular surface repopulation by peripheral corneal sphere cells

**Fig. 5:**
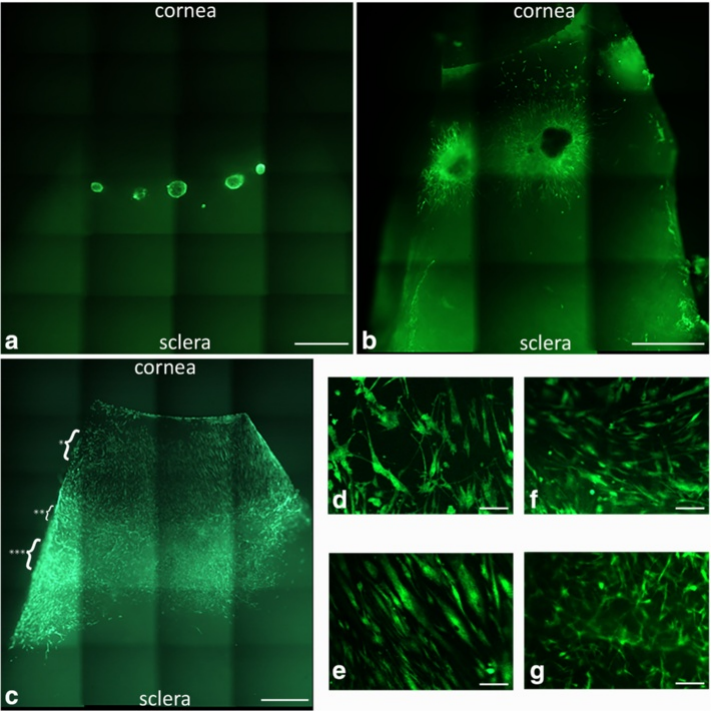
Peripheral corneal spheres implanted into corneosceral tissue repopulate the ocular surface. LIVE/DEAD® staining (*green*) of implanted spheres, 5× magnification, at 0 h post implantation **a** and 4 days post implantation **b** show cell migration from the spheres appearing as green streaks out from the sphere. At 7 days post implantation **c**, the entire corneal bed appears repopulated with live cells. Representative cells at the leading migratory edge of the corneal surface **d** and cells on the corneal surface (taken from the region indicated by * in c) **e** show differing morphology. Representative cells over the limbal region **f** and sclera **g** (taken from the regions indicated by ** and *** in **c** respectively) show a different cell migration pattern and morphology to that observed in the corneal tissue. Scale bar = 1000 μm for **a, b, e** and 100 μm for **c, d, f, g** (Colour figure online)

When spheres implanted in tissue at the limbal region (Fig. 5a) were cultured and imaged at 4 days, live migrating cells appeared outward from the sphere (Fig. 5b). At 7 days a centripetal cell migration pattern from the peripheral cornea out towards the direction of the central cornea (Fig. 5c) was observed. Cells displayed a preferential migration pattern onto clear cornea compared with sclera, seen as cells having migrated further on the corneal side of the implant compared with the scleral side. This was observed on more than 10 separate occasions from spheres derived from four different donors.

Cellular organization differed in cells observed at the cornea, limbus and sclera. In the limbal and scleral region, cells were elongated with a thin spindle appearance in comparison with cells at the corneal region (Fig. 5f, g) where they had a broader appearance. Cells at the leading migratory edge (Fig. 5d) displayed branching cellular processes.

A monolayer of migratory cells, with the longest axis of most cells aligning in the direction of the central cornea, was observed over the corneal surface (Fig. 5e). The alignments of the cellular axes gave the appearance of a relatively uniform migratory column over the cornea. There was an area of circumferentially oriented fibres in the limbal region seen as the longest horizontal axes of most cells aligning perpendicular to the direction of the central cornea (Fig. 5f). Over the sclera, however, cells occurred in multiple planes of focus whose longest axes did not uniformly align (Fig. 5g).

The number of cells populating the tissue was greater at day 7 post implantation than that which could be provided by sphere cell migration alone, indicating active cell proliferation coupled with the migration. The extent of the migration out towards the cornea was limited only by the size of donor tissue utilized, with cells present at the furthest corneal edge. Notably this extent of cell migration was not observed out towards the sclera (Fig. 5c).

### Cross-sectional and whole-mount immunocytochemistry

**Fig. 6:**
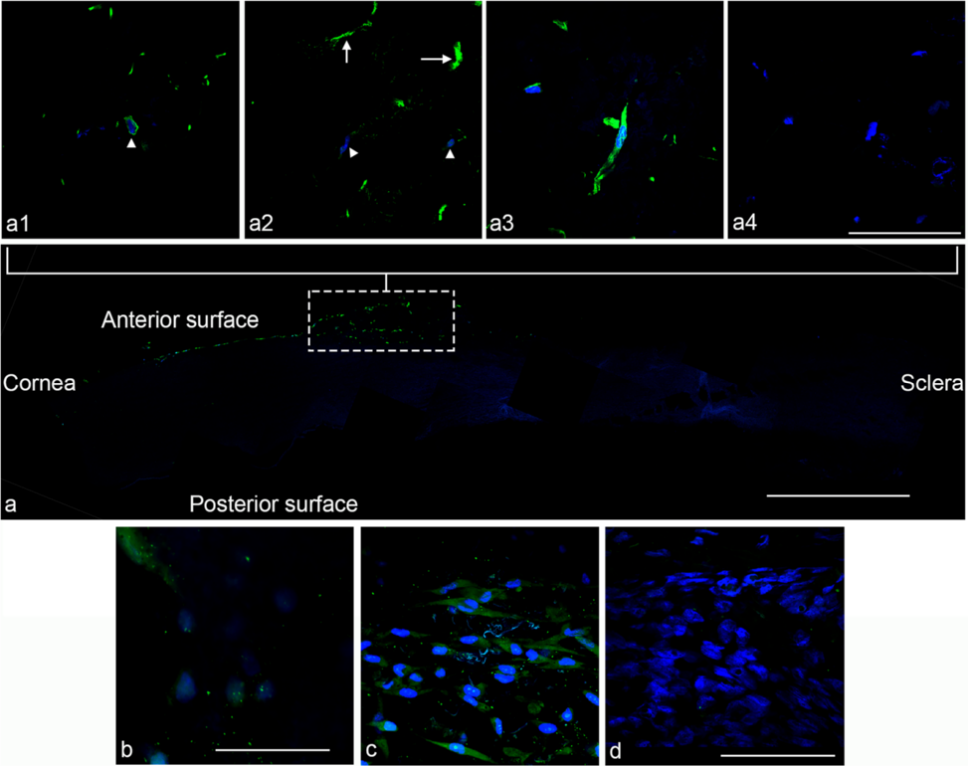
Immunocytochemistry of peripheral corneal spheres implanted into the limbal region (*dotted* region of interest **a**) of corneoscleral tissue and cultured for 14 days. Montage imaging showing monolayer of vimentin-stained cells (*green*) having migrated over the ‘anterior surface’ of the corneal bed **a**. Confocal imaging of immunostained cross-sections at 60× objective magnification shows ∆Np63α-positive staining (*green*) surrounding a cell nucleus **a1** (*arrowhead*), and laminin-positive staining **a2** showing clusters of strong green signals in tissue not associated with cells (*arrows*) and weaker positive signals close to or within the cell (*arrowheads*). Vimentin-positive green signals are seen associated with cell nuclei **a3** and a representative image of the secondary-antibody-only negative control shows no non-specific staining **a4**. Whole-mount sections show a positive signal for ABCG2 **b** and notch 1 **c**. Representative image of the secondary-antibody-only negative control **d** shows no green positive-staining. *Blue* signals represent DAPI staining of DNA within cell nuclei. Scale bar = 100 μm for **a1–a4**, 50 μm for **a** and 100 μm for **b, d** (Colour figure online)

In cross-section, sphere cells that had migrated over the cornea for 14 days formed a monolayer over the anterior surface (Fig. 6a). Towards the corneal periphery, however, cells had a less organized multilayered appearance (Fig. 6a, dotted region of interest). During the course of migration, cells remained superficial on the anterior surface and did not appear to migrate deep into the tissue substrate.

Immunostaining of cells implanted in tissue for 14 days revealed positive staining for the stem cell marker ∆Np63α (Fig. 6a1). Laminin staining (Fig. 6a2) revealed disorganized clusters of positive signals. Vimentin-positive cells (Fig. 6a3) were identified in cells migrating over the tissue. Additionally, whole-mount staining revealed the presence of the stem cell marker ABCG2 in a few cells (Fig. 6b) and the limbal basal epithelial marker, notch 1 (Fig. 6c). Immunostaining for keratocan using anti-keratin 3 yielded fluorescent signals equivalent to the negative control and therefore were not detected.

### Gene expression profile of sphere cells implanted into corneoscleral rims and cultured over time

**Fig. 7:**
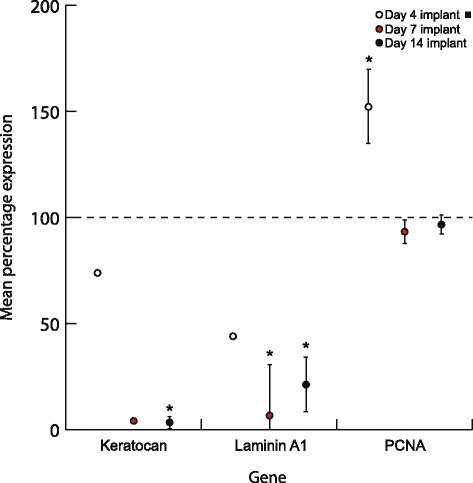
Expression of keratocan, laminin A1 and PCNA in corneal peripheral spheres implanted into corneoscleral tissue and cultured over 4, 7 and 14 days. All data are calibrated to non-implanted spheres (*dotted line*). Expression data (normalized to β-actin and GAPDH) are expressed as percentages of non-implanted spheres ± 1 standard error of spheres collected from three separate donors. Keratocan and laminin expression declined significantly post implantation. PCNA, however, was significantly elevated 4 days post implantation. There was a significant decrease in PCNA expression from day 4 to day 7. By days 7 and 14, PCNA expression is statistically equivalent to non-implanted sphere PCNA expression. *Black square* for day 4 implant indicates data available from only two donor sets due to tissue processing limitations. *p < 0.05. *PCNA* proliferating cell nuclear antigen

Expression data were calibrated against the expression value determined for non-implanted spheres. The keratocyte markers keratocan and laminin A1 were significantly reduced in implanted spheres 14 days post implantation compared with non-implanted spheres (Fig. 7). Mean keratocan expression was reduced by 97 % by day 14 (3.10 %) post implantation (*p* = 0.000). Mean laminin A1 expression significantly decreased by 93.3 % in day 7 implants (6.70 %) (*p* = 0.014) and remained at a depressed level at day 14 (21.16 %).

In contrast, mean proliferating cell nuclear antigen (PCNA) expression—a marker of cell proliferation—was increased significantly by 52.15 % at day 4 (*p* = 0.007), then decreased significantly (*p* < 0.05) by 58.70 % from day 4 to day 7 (93.37 %) (*p* = 0.004), returning to the level of the non-implanted sphere and remaining at this level to day 14 (96.67 %).

There was no significant difference in vimentin expression pre and post implantation due to high inter-donor variability in the expression of these markers (data not shown). Additionally, quantitative analyses on expression of the stem cell markers ABCB5, ABCG2 and p63α showed no significant change over time post implantation. Therefore it is unclear whether the stem cell population of the implanted spheres was maintained over the course of culture. Similarly, quantitation of the basal epithelial marker notch 1 and α-SMA, a marker of myofibroblastic transformation, showed no significant change over time post implantation.

## Discussion

### Peripheral corneal sphere characterization

Sphere isolation by the sphere-forming assay in serum-free conditions is well known to promote the survival of stem cells and progenitors, while discouraging the survival of more differentiated cells [24]. Recently, the *ABCB5* gene was convincingly purported as a putative limbal stem cell marker based on its co-expression with the long-standing putative limbal stem cell marker p63α, the reduced ABCB5-expressing cells in limbal stem cell deficiency patients as well as the ability for ABCB5-positive cells to restore an animal model of limbal stem cell deficiency [32]. Our results show that the cells of our peripheral corneal spheres also label positively for ABCB5, which not only provides further circumstantial evidence that ABCB5 may be a marker of human limbal stem cells but also confirms that spheres generated by our protocol are highly likely to be enriched with limbal stem and progenitor cells.

While being stem cell enriched, our peripheral corneal spheres are heterogeneous in nature with respect to cellular origin. We have previously shown exposure of spheres to collagen and differentiation medium produced both vimentin-positive and cytokeratin 3/12-positive cells [30]. We did not find extensive labelling for either of these markers within the spheres prior to exposure to these stimuli. Thus we propose that whilst the spheres contain cells of both stromal and epithelial origin, they are less differentiated than true epithelial or keratocyte cell types—but these differentiation markers are able to be stimulated, making it difficult to assess the relative proportions of cell populations within the sphere. However, through double immunostaining, Li et al. [33] utilized a similar cell isolation protocol to our laboratory and showed that, after the cell isolation process from tissue, 95 % of cells were vimentin-positive stromal cells while 5 % were pancytokeratin-positive epithelial cells. The corneal epithelial origins of our sphere cells is indicated by detection of basal epithelial markers notch 1 [34] and laminin [35] in immobilized spheres and keratin 3 in spheres given a migratory stimulus. Similarly, the corneal stromal origin of spheres cells is evidenced by keratocan [36] in immobilized spheres and vimentin in spheres given a migratory stimulus.

These results align with previous findings which assert that spheres are not simply clusters of functionally isolated cells, but an assemblage of cells within a matrix which mimics the basal corneal epithelium and stroma [37]. Although it may be argued that the extracellular matrix molecules may be from the cell extraction process as opposed to being produced by the cells themselves, laboratory observations showing the initial aggregation of cells followed by the growth of the sphere and our quantitative PCR findings showing a baseline expression of laminin and keratocan in spheres suggest an actively maintained microenvironment within the sphere. Moreover, the persistence of stem cells within spheres in culture over time should attest to the existence of an actively synthesized, functional extracellular matrix given the importance of the limbal niche environment in the maintenance of stem cell character. Collectively, we believe our characterization of these spheres confirmed their identity as stem and progenitor cell-enriched entities of epithelial and stromal origin that would facilitate testing of these as transplantable elements for corneal repair and regeneration.

### In-situ corneal repopulation

The implantation of peripheral corneal spheres requires an active process of cellular adherence to the human limbal substrate provided. Implanted spheres not only survived the implantation and culture process on their new substrate but were able to provide cells which migrated into the foreign limbal environment, thereby demonstrating a capacity for corneal repopulation in tissue. For the first time, we have shown that spheres implanted into the peripheral cornea and cultured over time are able to provide cells which extensively repopulate the entire available area of the corneal bed of the corneoscleral rim segment.

The observed centripetal cell migration pattern from peripheral cornea in the direction of the central cornea supports the well-established theoretical framework for corneal maintenance in vivo where stem cells from the limbus divide and provide centripetally migrating progeny which are responsible for corneal maintenance. Our results align with the longstanding view of corneal maintenance first proposed by Davanger and Evensen [1].

The manner of ocular surface repopulation over the cornea contrasts with the observations over sclera, suggesting that the regional difference in substrate composition exerts an effect on cell migration. Specifically, sphere cells which completely repopulated corneal tissue demonstrated a preferential migration in the corneal direction in comparison with the scleral direction as evidenced by cells having migrated a greater distance toward the central cornea from the site of implant. Cellular orientation may provide clues to explain this phenomenon. We observe that cells which have repopulated the corneal bed display a capacity to establish themselves in an anatomically appropriate orientation. A close examination of the orientation demonstrated by migrated cells revealed the regular, parallel arrangement of cells aligned with their long axes oriented toward the central cornea, while cells which repopulated the limbal region aligned circumferentially and the limited number of cells within the scleral region appeared in a quasi-random orientation with a lack of cellular alignment in a single direction. We believe that the stimuli for differential cell arrangements after implantation may be two-fold. Firstly, we hypothesize that spheres contain cells which are derived from the limbus and would like to reform a limbus or at least the limbal niche. To this aim, we are not surprised that the cells align differently on the corneal surface to those on the limbal surface and also appear repelled by the scleral surface. Secondly, there may also be residual structural signals for cell arrangement left on the decellularized tissue surface which further aids the seemingly pre-programmed nature of the cellular architecture that appears after implantation. This cellular orientation pattern may reflect the well-established orientation of collagen fibrils distributed within the ocular surface [38]. The limbus sports circumferentially oriented collagen fibrils [39, 40] while the fibrils of the sclera irregularly branch and intersect [38, 41]. The unique arrangement of the collagenous substrate of the cornea probably facilitates a greater extent of cell migration on the corneal side. Further mechanistic studies into the migratory properties of cells from spheres are limited primarily by the availability of human donor tissue for experimentation. Live cell observations in living tissue will be of value to deduce the effect of a biologically active sclera and conjunctiva.

Although the majority of implanted sphere-cell migration was towards the cornea, our results of cellular migration in the scleral direction appear to contribute to the mounting literary evidence surrounding the centrifugal pattern of injury response [26]. Chang et al. [42] demonstrated the centrifugal migration pattern of corneal cells in response to a corneal injury. Majo et al. [43] showed the potential for central corneal cells to participate in the response to injuries of the conjunctival epithelium. Here we show that sphere cells derived from the peripheral cornea and implanted into the limbus are able to elicit a scleral-directed migratory response, suggesting that the structure of the scleral surface, although biologically inactive, is not a barrier to cell migration in our experimental model.

### Sphere cell biology post implantation

Characterization of repopulated tissue post sphere implantation showed EDU positivity in cells from implanted spheres agreeing with PCNA quantification showing an initial significant increase in cell proliferation at day 4, followed by a significant decline from day 4 to day 7. These results align with our previous findings that the wound-healing response of spheres in vitro also shows a higher proliferative response by spheres at day 4 in comparison with day 7 and day 14 [44] where, as a result of direct compression injury, sphere cells displayed a more migratory rather than proliferative response at day 7 in comparison with day 4, which agrees with our current in-situ findings. The similarity in the cellular responses observed suggests that the implantation process is akin to a wounding process which generates a similarly reactive biological response by spheres. However, the proliferative response was expected to be higher than we observed. It may be that our initial time window of observation, 4 days post implantation, is possibly capturing the downward part of the PCNA trend since the wound-healing response post injury in mice was shown to begin as early as 24 h [45].

The downregulation of keratocan and the lack of its immunocytochemical detection reflects the loss of the keratocytic nature in preference for the proliferative/migratory response of implanted cells due to the combined effects of serum exposure as well as the migratory stimulus provided by the ocular surface. Similarly, laminin expression was quantitatively downregulated although there was immunocytochemical evidence of laminin, notch 1 and the stem cell markers ABCG2, ∆Np63α and notch1 post implantation, suggesting the possible maintenance of the basal limbal environment in prolonged culture and despite the predominantly proliferative phenotype post implantation.

Strongly positive vimentin staining of cells with immunochemistry could not be correlated with an increase in vimentin expression in the quantitative PCR data due to high donor variation in the expression of this gene. A larger source of donor tissue may be required to confirm the true trend in vimentin expression. The observed reduction in both stromal and epithelial cell markers post implantation may be indicative of an enhanced stem to progenitor cell response in the corneal repopulation observed over the 14-day time period resulting in the signal from already differentiated stromal and epithelial cells present in spheres being swamped by the increased signal from proliferating and migrating cells.

There appeared to be no significant change in the expression of stem cell, basal epithelial and myofibroblast markers (ABCB5, ABCG2, p63α, notch 1 and α-SMA) over the course of the implantation experiments. This may suggest that the original sphere cell features are being maintained over time but we cannot confirm whether true stem cell repopulation at the limbus has occurred. Further characterization of a larger donor set and longer experimental times may serve to establish the possibility of long-term stem cell repopulation by peripheral corneal spheres.

## Conclusion

Peripheral corneal spheres generated by the sphere-forming assay are stem cell enriched, possess properties of the native limbal microenvironment and can be successfully implanted into limbal tissue. This implantation of spheres results in cell migration and proliferation with evidence of cellular differentiation. There is preferential migration towards the cornea, the constraints of which were only the amount of corneal tissue surface provided. Viability of implanted spheres could be maintained beyond 14 days, indicating potential for prolonged restorative capacity. Collectively, these findings give the strongest evidence to date that peripheral corneal spheres could be developed into transplantable units for corneal repair in vivo and play a significant role in therapies targeting ocular surface regeneration and stem cell repopulation.

## Abbreviations

ABCB5: ATP-binding cassette sub-family B member 5
ABCG2: ATP-binding cassette sub-family G member 2
DAPI: 4′,6-diamidino-2-phenylindole
EDU: 5-ethynyl-2′-deoxyuridine
PCNA: proliferating cell nuclear antigen
